# Motor-Skill Learning Is Dependent on Astrocytic Activity

**DOI:** 10.1155/2015/938023

**Published:** 2015-08-04

**Authors:** Ragunathan Padmashri, Anand Suresh, Michael D. Boska, Anna Dunaevsky

**Affiliations:** ^1^Department of Developmental Neuroscience, Munroe-Meyer Institute, University of Nebraska Medical Center, Omaha, NE 68198, USA; ^2^Department of Radiology, University of Nebraska Medical Center, Omaha, NE 68198, USA

## Abstract

Motor-skill learning induces changes in synaptic structure and function in the primary motor cortex through the involvement of a long-term potentiation- (LTP-) like mechanism. Although there is evidence that calcium-dependent release of gliotransmitters by astrocytes plays an important role in synaptic transmission and plasticity, the role of astrocytes in motor-skill learning is not known. To test the hypothesis that astrocytic activity is necessary for motor-skill learning, we perturbed astrocytic function using pharmacological and genetic approaches. We find that perturbation of astrocytes either by selectively attenuating IP_3_R2 mediated astrocyte Ca^2+^ signaling or using an astrocyte specific metabolic inhibitor fluorocitrate (FC) results in impaired motor-skill learning of a forelimb reaching-task in mice. Moreover, the learning impairment caused by blocking astrocytic activity using FC was rescued by administration of the gliotransmitter D-serine. The learning impairments are likely caused by impaired LTP as FC blocked LTP in slices and prevented motor-skill training-induced increases in synaptic AMPA-type glutamate receptor *in vivo*. These results support the conclusion that normal astrocytic Ca^2+^ signaling during a reaching task is necessary for motor-skill learning.

## 1. Introduction

Astrocytes are in intimate structural relationship with synaptic contacts and are emerging as active players in development [1], function [2], and plasticity of synapses [3]. Astrocytes exhibit a large number of receptors for different neurotransmitters and the majority of these transmitters signal through G protein-coupled receptors (GPCRs) [4] that lead to IP_3_R2 mediated release of Ca^2+^ from intracellular stores [5]. The role of astrocytic Ca^2+^ signaling in modulating synaptic transmission, plasticity, and behavior is not fully clear. Following an increase in intracellular Ca^2+^, astrocytes release gliotransmitters [6, 7] such as glutamate, ATP, and D-serine that have been shown to regulate synaptic transmission and plasticity through a wide range of mechanisms [8–10]. D-serine is the endogenous ligand for the coagonist site of NMDARs and its contribution to synaptic plasticity has been shown in different brain regions [11–16]. Abolishing astrocytic GqGPCR Ca^2+^ signaling using IP_3_R2 KO impairs cholinergic-induced LTP in both hippocampus [17] and cortex [18] as well as nucleus basalis-induced stimulus-specific plasticity in visual cortical neurons [19]. In other studies though, modulation of IP_3_R2-mediated Ca^2+^-dependent activity had no effect on synaptic plasticity, neurovascular coupling, or several mouse behaviors [5, 20–24]. Owing to these conflicting reports, the role of astrocytes in modulation of synaptic plasticity and circuit function remains to be resolved.

To gain a better understanding of the role of astrocytic activity in modulation of behavior, we used pharmacological and genetic approaches to study learning in a motor-skill task. We find that mice with reduced IP_3_R2 mediated calcium signaling showed learning deficits. Furthermore, mice with metabolically inhibited astrocytes showed impaired learning that could be rescued with D-serine administration. We show that NMDA receptor-dependent LTP in the forelimb motor cortex was completely blocked in metabolically arrested astrocytes and this could be partially rescued with D-serine. Finally, motor-skill learning-induced increase in synaptic levels of GluA1 was blocked in these mice. We conclude that normal astrocytic activity and Ca^2+^ signaling during motor-skill training are necessary for normal learning to occur.

## 2. Materials and Methods

### 2.1. Animals

Unless indicated otherwise experiments were performed using C57BL/6 mice from Charles River Laboratories or from our breeding facility. The mice were kept on regular light/dark cycles throughout the procedures. All protocols were approved by the University of Nebraska Medical Center Institutional Animal Care and Use Committee.

### 2.2. Generation of Inducible Mutant IP_3_R2 (mIP_3_R2) Mice

IP_3_R2 floxed homozygous mice were obtained from Ken McCarthy's laboratory (UNC, Chapel Hill). To generate mutant IP_3_R2 mice, IP_3_R2^flox/flox^ mice were bred to mice expressing the tamoxifen-sensitive Cre-recombinase (Cre-ER) under the control of GLAST promoter (GLAST-CreER) [25]. Mice double heterozygous for the floxed and Cre-recombinase alleles were interbred to IP_3_R2^flox/flox^ mice to generate mice homozygous for the floxed IP_3_R2 allele and heterozygous for Cre-recombinase. Mice homozygous for IP_3_R2^flox/flox^ but null for the Cre-recombinase allele were designated as littermate control mice. Mice were maintained on the C57Bl/6 background. Mice were genotyped by PCR analysis using genomic DNA and primers specific to Cre-recombinase and the floxed IP_3_R2 allele.

### 2.3. Tamoxifen Treatment

Tamoxifen was dissolved in corn oil to obtain a 10 mg/mL solution. For all experiments mice were injected intraperitoneally with a tamoxifen dosage of 100 mg/kg body weight for 8 days on a daily basis. Injections were performed at 3 weeks of age. Experimental cohorts were always injected at the same time. Control mice were homozygously floxed but these mice either lacked CreER expression and were injected with tamoxifen (tam control) or expressed CreER and received corn oil (vehicle control). Experiments were performed one-month posttamoxifen injections.

### 2.4. ICV Cannulation

Guide cannulas were implanted to the mouse brain when repeated injections into the cerebral lateral ventricles (intracerebroventricular injections) were performed. The mice were anaesthetized with ketamine/dexdomitor cocktail (100 mg/mL and 0.5 mg/mL, resp., 2.5 mL/Kg) and placed in a stereotaxic frame (Stoelting). A 26-gauge stainless steel guide cannula (Plastics One) was implanted into the brain (0.3 mm posterior, and 1.2 mm lateral to bregma, 2.4 mm below the surface of the skull). The coordinates were chosen so that the protruding injection cannula reached the lateral ventricle upon injection. The guide cannula was held in position by a combination of glue and cyanoacrylate gel. Mice were allowed 3-4 days of postoperative recovery before the injections. ICV injections (2 *μ*L) were carried out during a short period of anesthesia with ketamine/dexdomitor cocktail. Saline or Fluorocitrate was injected through stainless steel injector cannula (33-gauge) that fits into the guide cannula. A 5 mL Hamilton syringe was connected to a plastic tube and used for injection. Mouse was injected with the reversal agent Antisedan (atipamezole hydrochloride 5 mg/mL, at 0.2 mL/mL of Ketamine/Dexdomitor cocktail used, IP).

The fluorocitrate (FC) solution for ICV injection was prepared [26] as follows: 8 mg of D,L-fluorocitric acid, barium salt (Sigma-Aldrich) was dissolved in 1 mL of 0.1 M HCl. Two to three drops of 0.1 M Na_2_SO_4_ were added to precipitate Ba^2+^. Two milliliters of 0.1 M Na_2_HPO4 was added and the suspension was centrifuged at 1,000 g for 5 min. The supernatant was diluted with 0.9% NaCl to the final concentration and the pH was adjusted to 7.4.

The positioning of cannula implanted in each mouse was verified after completion of motor-skill training. The mice were anaesthetized and a small volume of fast green dye was injected through the cannula. The mice were transcardially perfused with 4% paraformaldehyde in 0.1 M phosphate buffer (pH 7.4) and the brains were postfixed in that solution at 4°C. The brains were sectioned at 150 *μ*m thickness on a vibratome (Leica). The sections were mounted on glass slides and viewed under an upright microscope (Nikon) to verify if the implanted cannula had reached the lateral ventricle. Mice in which the cannula did not reach the lateral ventricle were excluded from the study.

### 2.5. MRS Method

For MRS experiments a small craniotomy was made 0.9 mm posterior and 1.2 lateral to Bregma and FC was injected using a glass micropipette and a picospritzer. Control mice and mice injected with FC (3 hours after injection) were anesthetized by inhalation of 1-2% isoflurane in a nitrous oxide/oxygen mixture. MRI/MRS data were obtained using a 7 Tesla/16 cm Bruker Pharmascan (Karlsuhe, Germany), an actively decoupled 72 mm volume coil transmitter, and a laboratory built 1.25 × 1.5 cm surface coil receiver. Reference coronal mouse brain images were obtained using a RARE T_2_ weighted sequence with acquisition parameters of 20 mm field-of-view, a 256 × 256 acquisition matrix, first echo time of 12 ms, effective echo time of 36 ms, RARE factor = 8, repetition time of 4200 ms, and two averages for a total acquisition time of 3.3 min. Single voxel localized spectra were acquired using point resolved spectroscopy (PRESS) with outer volume suppression and high bandwidth pulses to optimize sequence performance. Spectra were acquired from a region spanning the motor cortex with a repetition time of 4 seconds, echo time of 50 ms, and 576 averages for a total scan time of 38.4 min. Single-scan-localized unsuppressed water signals were acquired as a reference for metabolite quantification. Spectroscopic data were processed by removal of residual water signal using the HLVSD filter. ^1^H MRS were curve fit in the time domain using the QUEST algorithm [27] with a linear combination of spectra (basis set) obtained from phantoms scanned using the same pulse sequence as the* in vivo* acquisitions. Normalized signal amplitudes were presented as a fraction of total signal.

### 2.6. Motor-Skill Training

Motor-skill training was performed on 5-week-old male C57Bl/6 mice for studies using FC and on 8-9-week-old IP_3_R2 mutant mice and their littermate controls. Mice were food restricted prior to training, maintaining them at 85% of their free-feeding weight. All training was performed between 3 and 7 PM. For training, mice were placed in a cage with a thin slit on the front wall. Mice had to learn to reach through the slit with the preferred forelimb and grasp and retrieve individual food pellets. The initial pretraining session was used for forelimb preference determination. Pellets were then placed on either side depending on the preferred forelimb. Mice had one training session per day that lasted 30 minutes or 100 reaches. Motor-skill performance was quantified by the success rate (pellets retrieved/total attempted). The experimental identity of the mice was not known during training.

ICV injections with saline or fluorocitrate were done 4 hours before motor-skill training. On days 6 and 7, mice were injected with D-serine (800 mg/kg, IP) 30 minutes before motor-skill training.

### 2.7. Live Slice Preparation

Acute coronal slices (300 *μ*m for slice imaging and 400 *μ*m for slice electrophysiology) containing the forelimb area of M1 were prepared in an ice-cold slicing solution containing (in mM) 126 NaCl, 3 KCl, 1.25 NaH_2_PO_4_, 4 MgSO_4_, 2 CaCl_2_, 26 NaHCO_3_, and 10 dextrose. Slices were transferred to a submersion chamber containing artificial CSF (ACSF, mM) 126 NaCl, 3 KCl, 1.25 NaH_2_PO_4_, 1 MgSO_4_, 2 CaCl_2_, 26 NaHCO_3_, and 10 dextrose and the solution was continuously bubbled with 95% O_2_/5% CO_2_. Slices were allowed to rest for at least 60 minutes before recording. For recordings, slices were transferred to submersion type recording chamber (Warner) and superfused at room temperature with ACSF saturated with 95% O_2_/5% CO_2_.

### 2.8. Slice Electrophysiology

Recordings were made from coronal cortical slices containing the forelimb representation of the M1. Tungsten bipolar stimulation electrodes were positioned in upper layer 2/3, 1.5–1.8 mm lateral to the midline [28], and recording electrodes were placed 400 *μ*m medial to the stimulation electrode. Extracellular field potentials (FPs) were evoked by 0.2 ms pulses at 0.033 Hz. Peak amplitudes of the negative-going FP were used as a measure of synaptic strength [29]. To examine synaptic strength, input-output curves for a range of stimulation intensities (multiples of threshold intensity defined as the intensity that produced a response of 0.2 mV) were recorded. LTP was induced after establishing a 10-minute period of stable response amplitudes using stimulation intensity that elicited 50% maximum response amplitude. Theta burst stimulation (TBS) was applied after a 3-minute bath application of bicuculline methiodide (10 *μ*M). The LTP induction protocol was five TBS delivered every 10 s. Each TBS consisted of 10 bursts (1 burst = 5 pulses at 100 Hz) delivered at 5 Hz.

To assess the effects of fluorocitrate (FC) on LTP, extracellular field potentials were recorded from slices perfused for 20 minutes with FC (100 *μ*M) alone or FC (100 *μ*M) supplemented with D-serine (100 *μ*M) or FC (100 *μ*M) with AP-5 (50 *μ*M) supplemented with D-serine (100 *μ*M) prior to commencing with recordings.

### 2.9. Dye Loading and Imaging in Slices

Slices were loaded at room temperature with fluo-4 AM (5 *μ*M, Molecular Probes), 0.04% Pluronic F-127, and SR101 (10 *μ*M, Sigma-Aldrich) for 40 minutes and then transferred to dye free ACSF for at least 30 minutes. Simultaneous monitoring of multiple layer 1 astrocytes was carried out in frame scan mode at 1 Hz at image resolution of 256 × 256. Time-lapse imaging was carried out; image series consisting of 300 frames were collected.

In order to assess the Ca^2+^ activity of astrocytes in the presence of FC, spontaneous Ca^2+^ changes before and after perfusion of FC (100 *μ*M) in the slices were compared. Following baseline imaging in regular ACSF, slices were perfused for 30 minutes with FC and then the same positions were reimaged in presence of FC.

To assess the efficiency of the Cre-mediated recombination, we performed Ca^2+^ imaging on bulk loaded coronal slices containing the M1 from controls (vehicle control and tamoxifen controls) and mutant IP_3_R2 mice. Time-lapse imaging was carried out to monitor spontaneous Ca^2+^ oscillations followed by reimaging of some of the same positions with perfusion of ATP (100 *μ*M).

We imaged with a two-photon microscope (Moving Objective Microscope, Sutter) attached to a Ti : sapphire laser (Chameleon Vision II, Coherent), using a 60x water immersion objective (1.0 NA, Nikon). Excitation wavelength of 800 nm with power measured at the back aperture at 10–30 mW was used. Two-channel detection of emission wavelength was achieved by using a 565 nm dichroic mirror and two external photomultiplier tubes. A 535/50 bandpass filter was used to detect fluo-4 AM emission wavelength, and a 610/75bandpass filter was used to detect SR101. For imaging, we used ScanImage software [30] written in MATLAB (The MathWorks).

### 2.10. Image Analysis

Imaging data was analyzed using ImageJ software. Regions of interest (ROIs) were placed around astrocyte soma. Fluorescence was averaged over ROIs placed and expressed as relative fluorescence changes (Δ*F*/*F*) after subtraction of background fluorescence from a neighboring region. Ca^2+^ transients of astrocyte were detected when fluorescence intensity reached higher than 2 SD value of baseline fluorescence intensity.

The following parameters were analyzed to assess the Ca^2+^ activity in the presence of FC: percent active astrocytes, the mean number of spontaneous events per astrocyte, and the average peak value of the Ca^2+^ oscillations. We analyzed the percent active cells and percentage of cells responding to ATP to assess the spontaneous Ca^2+^ changes and ATP-induced Ca^2+^ signals in control and mutant IP_3_R2 mice. For astrocyte soma analysis, an astrocyte that exhibited at least one spontaneous Ca^2+^ oscillation during the imaging session was referred to as an active astrocyte. The ratio of the number of active astrocytes and SR101 labeled cells in every imaging field was used to analyze the percent active astrocytes based on soma Ca^2+^ measurements. Analysis was performed on raw unprocessed images and for presentation purposes images were despeckled.

### 2.11. Confocal Imaging

Confocal imaging was performed on a Zeiss, LSM 700, using a 40 × 1.4 N.A oil lens. Images were collected in layer 1 of the forelimb M1 region at 512 × 512 pixels with pixel size of 0.18 *μ*m and *Z* step of 0.20 *μ*m, at 8 bit with 488 nm laser.

Dendritic spine analysis was done using ImageJ software. Dendritic segments analyzed were 20–80 *μ*m in length. Within each image, 1–3 dendrites were analyzed.

### 2.12. GluA1 Measurement in Synaptosomes

Four hours following an ICV injection of saline or 50 *μ*M FC, mice were trained on the forelimb-reaching task. On the following day, the M1 forelimb regions from both hemispheres were dissected out from two coronal slices (750 *μ*m). The tissue was homogenized in sucrose media (0.32 M sucrose, 10 mM HEPES, and 1 mM EDTA) using a hand held homogenizer. The homogenate was spun at 1,500 rpm for 10 min at 4°C. The supernatant was then spun at 13,300 rpm for 15 min at 4°C, to separate the synaptosomal fraction. This was resuspended in ACSF and protein concentration was estimated. Synaptosomes were lysed in RIPA buffer (50 mM Tris.HCl, 25 mM NaCl, 0.1% SDS, 0.5% Na deoxycholate, 1% Triton X 100, and 0.5 M EDTA). Proteins were separated on 4–15% Bio-Rad Mini-Protean TGX Precast SDS–PAGE gels and transferred to polyvinyl difluoride membranes. The membranes were probed with anti-GluA1 (1 : 1,500, Millipore) and anti-GAPDH (1 : 4,000, Cell Signaling). The membranes were incubated with horseradish peroxidase-conjugated secondary antibodies (anti-rabbit 1 : 2,500), and bands visualized using a Cell Biosciences FluorChem HD2 system.

### 2.13. Statistics

Data are reported as mean ± s.e.m. Normal distribution was tested using Kolmogorov-Smirnov test and variance was compared. Analysis was done either using two-sided unpaired Student's *t*-test (with a Bonferroni correction for multiple comparisons) or with one-way ANOVA with the Bonferroni method for* post hoc* multiple comparisons or two-way ANOVA with Sidak's method for post hoc multiple comparisons. Data were analyzed using the GraphPad Prism.

## 3. Results

Whether astrocytic activity is necessary for synaptic plasticity and learning is still debated. Here we used genetic and pharmacological methods to perturb astrocytes to understand the role of astrocytes in learning.

### 3.1. Reduced Motor-Skill Learning in Mutant IP_3_R2 Mice

**Figure 1:**
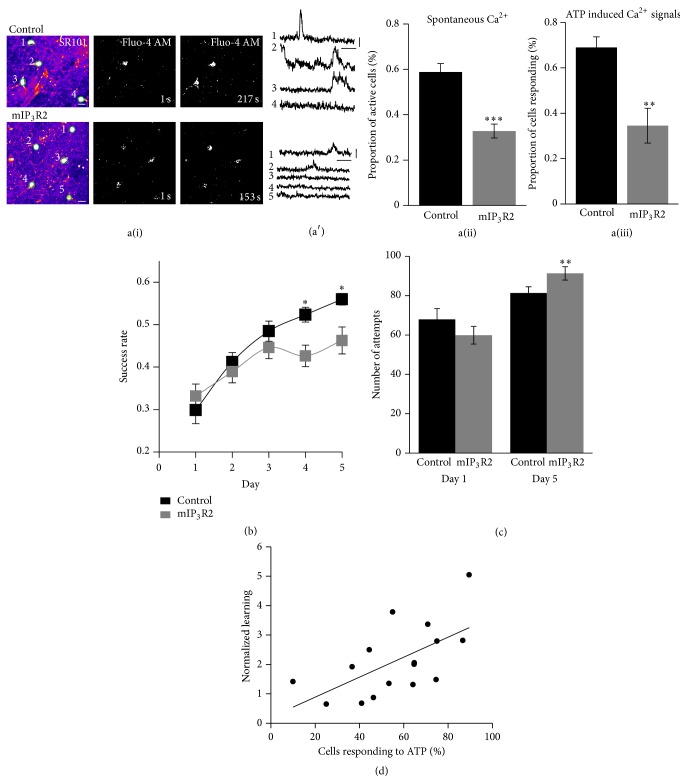
IP_3_R2 mediated Ca^2+^ signaling is necessary for motor-skill learning. a(i) Dual loading of acute M1 slices with SR101 (left) and fluo-4 AM (right) in control (top panel) and mIP_3_R2 (bottom panel) mice. ROIs of astrocyte soma are outlined and numbered. Time-lapse images of the fluo-4 channel for control and mIP_3_R2 KO mice showing spontaneous Ca^2+^ transients in soma. Scale bar: 10 *μ*m. (a′) Spontaneous Ca^2+^ transients in soma in control and mIP_3_R2 mice. Scale bar: 50 s, 100% Δ*F*/*F*. a(ii) Quantification of the proportion of active cells with spontaneous Ca^2+^ transients in soma in control and mIP_3_R2 slices. a(iii) Quantification of ATP-induced Ca^2+^ transients in control and mIP_3_R2 slices. (b) Motor-skill learning curves for control and mIP_3_R2 mice. mIP_3_R2 mice show a deficit in motor-skill learning. Repeated measures two-way ANOVA revealed a significant interaction between genotype and training factors (*P* = 0.0055). (c) Number of reaching attempts made on day 1 and day 5 in control and mIP_3_R2 mice. (d) Relationship between ATP-induced Ca^2+^ transients and learning (success rate on day 4/success rate on day 1). ^*^ *P* < 0.05, ^**^ *P* < 0.01, and ^***^ *P* < 0.0001.

In our first approach, we tested motor-skill learning in mutant IP_3_R2 (mIP_3_R2) mice. To avoid potential developmental changes we crossed the IP_3_R2^flox/flox^ mice with GLAST-Cre-ER mice [25], induced recombination at 3-4 weeks, and tested the mutant and littermate control mice at 8-9 weeks of age. To assess the recombination efficiency and the loss of IP_3_R2 in astrocytes, we performed Ca^2+^ imaging on bulk loaded coronal slices containing the forelimb M1 from control and mIP_3_R2 mice (Figure 1a(i)). We detected a 44% reduction in the number of astrocytes that exhibit spontaneous Ca^2+^ transients in slices from mIP_3_R2 mice (Figure 1a(ii), control: 58.78 ± 3.7% *n* = 14 slices from 11 mice, mIP_3_R2: 32.81 ± 3.08%, *n* = 15 slices from 14 mice; *P* < 0.0001, *t*-test). Similarly, there was a 50% reduction in the number of astrocytes that exhibited a Ca^2+^ transients in the presence of 100 *μ*M ATP (Figure 1a(iii), control: 69.02 ± 4.69%, *n* = 10 slices from 9 mice; mIP_3_R2: 34.56 ± 7.68%, *n* = 8 slices from 8 mice; *P* = 0.001, *t*-test). The data indicate a partial reduction of astrocytic Ca^2+^ signaling in the mIP_3_R2 mice.

To determine the role of astrocytic Ca^2+^ signaling in motor-skill learning we tested the mice on the forelimb reaching task. Performance was identical in both control groups (Tamoxifen and vehicle controls) and these were therefore pooled (Supplementary Figure  1, available online at http://dx.doi.org/10.1155/2015/938023). Although the success rate of both the IP_3_R2 mutant and littermate control mice increased over subsequent days of training, the mutant achieved a lower success rate on the fourth and fifth day of training (Figure 1(b)). Significant differences were found on day 4 (Control: 0.52 ± 0.02, *n* = 21 mice; mIP_3_R2: 0.43 ± 0.03, *n* = 21 mice, Two-way ANOVA,* post hoc* test, *P* = 0.035) and day 5 (Control: 0.56 ± 0.01; mIP_3_R2: 0.46 ± 0.03,* post hoc* test *P* = 0.035, Figure 1(b)). The success rate on the first day of training (Control: 0.3 ± 0.03; mIP_3_R2: 0.33 ± 0.03, *P* = 0.8) was not different between the genotypes. Moreover, the mIP_3_R2 mice did not make fewer reaching attempts than the control mice (day 1, Control: 67.95 ± 5.46; mIP_3_R2: 59.86 ± 4.49, *P* = 0.259; day 5, Control: 81.29 ± 3.22; mIP_3_R2: 91.29 ± 3.39, *t*-test, *P* = 0.009Figure 1(c)). These data indicate that mIP_3_R2 mice displayed a mild learning impairment that is not due to basic motor function deficit or lack of motivation.

We also wanted to determine if there was a relationship between astrocyte Ca^2+^ and behavior. For a subset of mice (both control and mIP_3_R2) following behavioral testing on the forelimb reaching task, slices were prepared and ATP-evoked astrocyte Ca^2+^ activity was measured. We performed the correlation with proportion of cells exhibiting ATP evoked Ca^2+^ activity rather than spontaneous Ca^2+^ activity in order to more accurately correlate behavior with ability of astrocytes to generate Ca^2+^ signals. We found a positive correlation between ATP evoked astrocyte Ca^2+^ and learning (Figure 1(d), *P* = 0.01, *r* = 0.61) suggesting that there is a link between astrocyte Ca^2+^ and the motor learning deficit in the mIP_3_R2 mice. Our findings therefore suggest that astrocytic Ca^2+^ signaling is involved in modulating behavior.

### 3.2. Impaired Motor-Skill Learning in Mice with Metabolically Inhibited Astrocytes Is Reversed with D-Serine

**Figure 2:**
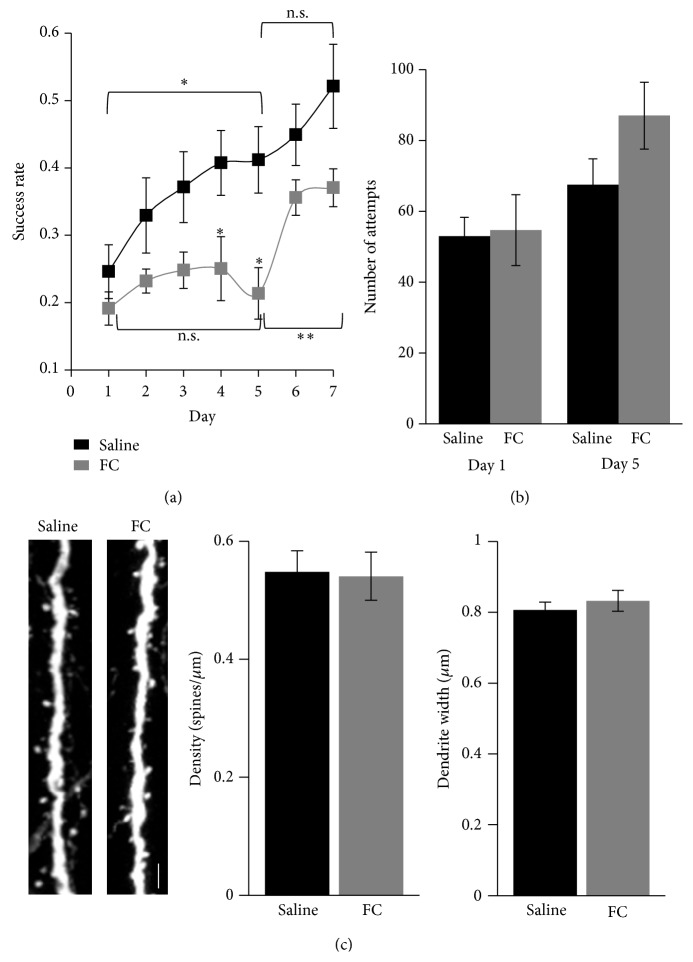
Astrocytic activity is necessary for motor skill learning. (a) Motor skill learning curves for saline and FC injected mice. Two-way ANOVA revealed a significant effect with training (*P* = 0.0001) and treatment (*P* < 0.0001). FC injected mice show a deficit in motor skill learning that is rescued by D-serine on days 6 and 7. (b) No difference was detected between number of reaching attempts made on day 1 and day 5 with FC injection. (c) Representative images of dendritic spines in saline and FC injected conditions. Scale bar: 3 *μ*m. FC injection does not alter the dendrite width and spine density. ^*^ *P* < 0.05 and ^**^ *P* < 0.01.

As a second approach to determine the role of astrocytic activity in motor-skill learning we used an astrocyte specific metabolic inhibitor fluorocitrate (FC). FC inhibits the TCA cycle in glial cells by blocking aconitase [31] with significant reductions in aconitase activity and ATP levels in the cortex four hours after intraventricular injection [32]. FC effects* in vivo* are known to be reversible with maximal effects observed four hours after an intrastriatal injection [26] and six hours after intravitreal injections [33]. Using Magnetic Resonance Spectroscopy (MRS) (Supplementary Figure  2) we detected a reduction in cortical glutamate levels four hours following intracerebroventricular (ICV) injection of 50 *μ*M (100 pmol) FC (control: 0.27 ± 0.01, *n* = 7; FC: 0.18 ± 0.02, *n* = 6  *t*-test, *P* = 0.013). Reduced levels of glutamate would be expected because of the known role of astrocytes in the glutamate/glutamine cycle [34, 35]. We therefore performed an ICV injection of FC or saline daily, 4 hours prior to motor-skill training (see Supplementary Figure 3). Strikingly, mice injected with 10–50 *μ*M (20-100 pmol) of FC failed to improve in the motor-skill task (saline day 5 success rate: 0.41 ± 0.05, *n* = 8 mice; FC day 5: 0.21 ± 0.04, *n* = 6 mice, two-way ANOVA* post hoc* test: *P* = 0.024, Figure 2(a)). The impaired learning was not due to the cannula insertion because saline injected mice displayed normal learning curves (day 1: success rate of 0.25 ± 0.04, day 5: success rate of 0.41 ± 0.05, *n* = 8 mice, *t*-test, *P* = 0.02, Figure 2(a)), as did mice injected with 10–50 nM (20–100 fmol) of FC (Supplementary Figure  4). The learning impairment was not due to reduced motor activity since the number of reaching attempts made by FC injected mice was not different from saline injected mice (day 1 saline: 53 ± 5.31, FC: 54.67 ± 9.99, *t*-test, *P* = 0.877; day 5 saline: 67.5 ± 7.33, FC: 87 ± 9.44, *t*-test, *P* = 0.081, Figure 2(b)). We also determined that 50 *μ*M (100 pmol) FC injection did not have a detrimental effect on neuronal structure by imaging dendrites in layer 1 of forelimb M1 region. We observed no difference in the density of dendritic spines (saline: 0.55 ± 0.04 spines/*μ*m, *n* = 27 images from 2 mice; FC: 0.54 ± 0.04 spines/*μ*m, *n* = 23 images from 2 mice; *P* = 0.68, Figure 2(c)) or dendrite width (saline: 0.81 ± 0.02 *μ*m; FC: 0.83 ± 0.03 *μ*m; *P* = 0.57) in the apical tufts (layer 1) of layer 5 pyramidal neurons in the forelimb M1 region. These results suggest that normal astrocytic activity during motor-skill training is necessary for motor-skill learning.

Since NMDA receptor dependent LTP-like mechanism is thought to be involved in motor-skill learning [36], we next asked if the gliotransmitter D-serine, which acts as a coagonist for NMDA receptors and regulates neurotransmission and synaptic plasticity, can reverse the effects of FC on motor-skill learning. On the 6th and 7th days of FC or saline injection, mice were administered with 800 mg/Kg D-serine intraperitoneally, 30 minutes prior to motor-skill training (Supplementary Figure 3). We found that the learning impairment with FC was rescued by coadministration of D-serine (day 7: success rate of 0.37 ± 0.03, day 5 versus day 7 *t*-test, *P* = 0.008, Figure 2(a)). In the saline injected mice we saw a trend towards increased success rate when D-serine was administered (day 7: success rate of 0.52 ± 0.06, day 5 versus day 7 *t*-test, *P* = 0.191). These results indicate that increased levels of D-serine at a time of motor-skill training result in learning even in conditions with reduced astrocytic activity.

### 3.3. LTP Is Blocked by FC in Slices and* In Vivo*

**Figure 3:**
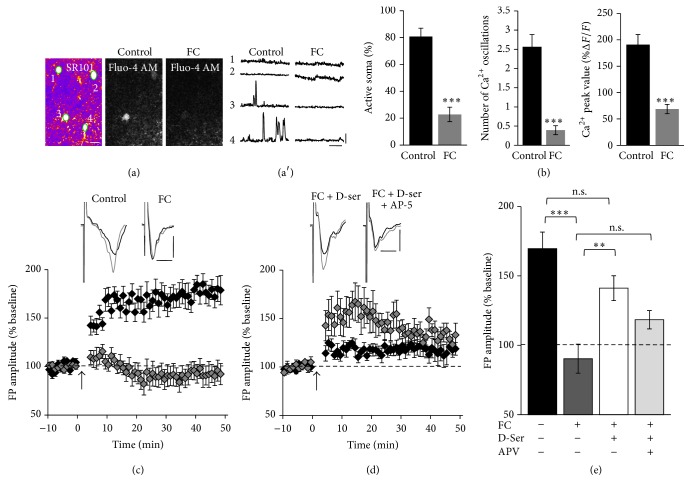
Fluorocitrate blocks astrocytic Ca^2+^ transients and LTP in slices. (a) Dual loading of acute M1 slices with SR101 (left) and fluo-4 AM (right) in control and after perfusion of FC. ROIs of astrocyte soma are outlined and numbered. Time-lapse images of the fluo-4 channel for control and after FC conditions. Scale bar: 10 *μ*m. (a′) Spontaneous Ca^2+^ transients in soma in control and FC conditions. Scale bar: 75 s, 100% Δ*F*/*F*. (b) The percent of active soma, the number of Ca^2+^ transients, and the peak value of the Ca^2+^ transients significantly decreases after treatment with FC. (c) Average time course of the change in FPs in M1 slices after TBS (indicated by arrow) shown for control and FC conditions. Incubation of slices with FC blocks LTP. Representative FPs (average of 5 traces) during baseline condition and 20–30 min after TBS are shown as black and gray traces. Scale bar: 0.4 mV, 4 ms. (d) Average time course of the change in FPs shown for FC coapplied with D-serine, FC coapplied with D-serine and D-AP5. D-Serine partially rescues LTP. (e) Summary of LTP experiments with FPs measured 20–30 min after TBS relative to baseline. ^**^ *P* < 0.01 and ^***^ *P* < 0.0001.

To further investigate the possibility that FC blocks learning by interfering with an LTP-like mechanism we next determined if FC blocks LTP in slices containing the motor cortex. In acute brain slices, FC (100 *μ*M) blocked Ca^2+^ transients in astrocytes (Figures 3(a) and 3(b)). The percent active astrocytes (control: 80.78 ± 6.13%, FC: 22.78 ± 5.42%, *t*-test, *P* < 0.0001), the number of Ca^2+^ transients (control: 2.57 ± 0.32, FC: 0.39 ± 0.12, *t*-test, *P* < 0.0001), and the peak value of Ca^2+^ transients (control: 190.97 ± 18.87, FC: 68.89 ± 8.89, *t*-test, *P* < 0.0001) were significantly reduced after incubation in 100 *μ*M FC (*n* = 15 imaged fields from 3 slices in control and FC conditions). To test if LTP is impaired under these reduced Ca^2+^ signaling conditions, field potentials were recorded in layer 2/3 in response to theta burst stimulation (TBS) of the horizontal connections [29]. While in control slices a robust LTP could be induced, it was completely blocked by 100*μ*M FC (Figure 3(c), control: 169.66 ± 11.92%, *n* = 8 slices from 7 mice; FC: 90.34 ± 10.5%, *n* = 8 slices from 8 mice, one-way ANOVA, *P* < 0.0001,* post hoc* test control versus FC, *P* < 0.0001). LTP was partially restored when 100 *μ*M of the gliotransmitter D-serine, which acts as a coagonist for NMDA receptors, was coapplied with FC (Figure 3(d), FC+D-ser: 141.19 ± 8.97%, *n* = 8 slices from 7 mice,* post hoc* test, FC versus FC + D-Serine, *P* = 0.0037). The effect of FC on LTP was not restored when D-serine was applied in the presence of the NMDA receptor antagonist AP-5 (FC+D-ser+AP-5: 118.43 ± 6.61%, *n* = 8 slices from 5 mice,* post hoc* test FC versus FC+D-ser+AP-5, *P* = 0.186). These results suggest that as in other brain regions [13, 14], LTP in the motor cortex is dependent on astrocyte derived D-serine.

**Figure 4:**
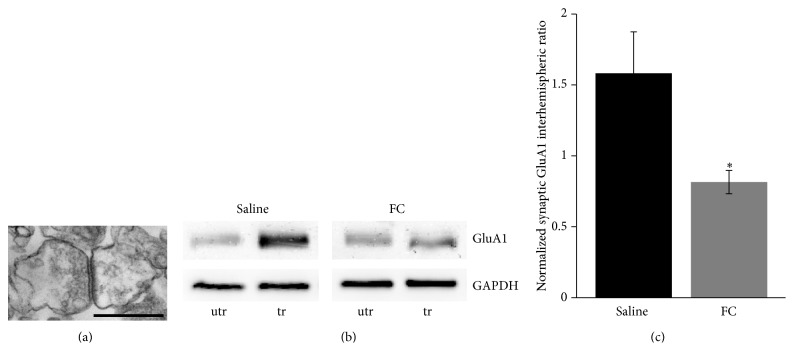
Astrocyte function is necessary for motor-skill learning induced synaptic GluA1 insertion. (a) An example electron micrograph demonstrating the presence of synaptosomes. Scale bar: 1 micrometer. (b) Western blots of synaptic GluA1 from forelimb M1 regions of untrained (utr) and trained (tr) hemispheres of 1 day trained mice following ICV injections with saline or FC. (c) Interhemispheric ratios (trained/untrained) of synaptic GluA1. ^*^ *P* < 0.05.

Finally, to determine if FC interferes with an LTP-like mechanism* in vivo*, we asked if translocation of glutamate receptor subunit 1 (GluA1) into synapses that is known to occur with LTP [37] and with motor-skill learning [38] is blocked by FC. We prepared synaptosomes (Figure 4(a)) from the forelimb M1 region of both hemispheres of 1 day trained mice and measured GluA1 levels (Figures 4(b) and 4(c)). While in saline injected mice there was an increase in the levels of GluA1 in the trained hemisphere (contralateral to the trained forelimb) there was no such increase in FC (10 *μ*M, 20 pmol) injected mice (saline: interhemisphere ratio 1.58 ± 0.29, *n* = 9, FC: interhemisphere ratio 0.81 ± 0.08, *n* = 7, *t*-test, *P* = 0.033). These data suggest that absence of optimal astrocytic function* in vivo* prevents training induced synaptic changes and might mediate the impaired learning.

## 4. Discussion

Our findings of reduced astrocytic activity resulting in deficits in motor-skill learning, LTP in slices, and synaptic AMPAR insertion with motor-skill training and that D-serine, the NMDAR coagonist, can rescue both LTP and learning and provide support to the hypothesis that astrocytic Ca^2+^ signaling and release of D-serine is necessary for the LTP-like mechanism that is mediating motor-skill learning.

Although cognitive impairments have been reported when astrocytes are perturbed through genetic [39–42] or pharmacological manipulations [43–45], astrocytic function has not been previously implicated in motor-skill learning. Here, we show that both chronic but partial disruption of IP_3_R2 mediated Ca^2+^ signaling in astrocytes as well as acute disruption of astrocytic activity with the astrocyte specific metabolic inhibitor FC lead to impaired learning on a motor-skill task.

The activation of IP_3_R2 in astrocytes is primarily responsible for increases in astrocyte Ca^2+^ [5, 18], although the involvement of other mechanisms in generation of Ca^2+^ signals have recently been shown [10, 46–48]. We find that mIP_3_R2 mice exhibit a mild learning impairment in the motor-skill learning task that is consistent with only a partial loss of IP_3_R2-dependent Ca^2+^ signaling in these mice. It is possible that in the complete KO mice, the cognitive deficit would be more pronounced, as is the case with mice that were injected with the astrocytic metabolic inhibitor FC. In our study the loss of IP_3_R2 in astrocytes was induced after brain development was complete. Therefore, the changes we observe are not likely to be due to abnormal neuronal circuit formation. Other studies also show evidence for behavioral impairments when IP_3_R2-dependent Ca^2+^ signaling is altered [49, 50] but see [23]. For example, expression of an inducible IP_3_ absorbent “IP_3_ sponge” in astrocytes was found to attenuate IP_3_R-mediated Ca^2+^ signaling and these mice were found to exhibit impaired spatial reference memory and reduced contextual fear memory [49]. In a full germ-line IP_3_R2-KO mice, the lack of IP_3_R2 was shown to affect astrocytic ATP release and induce depressive-like behaviors [50].

In this study, we also used FC that has been used in several studies as a selective inhibitor of glial cell metabolism [32, 33, 51, 52]. Unlike in the mutant IP_3_R2 mice, the astrocytic impairment in FC injected mice is transient and is timed to be maximal during the motor-skill training sessions. Inhibition of glial aconitase by FC leads to accumulation of citrate that acts as a Ca^2+^ chelator [31]. The reduction in spontaneous Ca^2+^ activity in astrocytes that we observe with FC could be a result of citrate accumulation although other effects on astrocyte activity are likely. Nevertheless, our studies with the genetic and pharmacological manipulations of astrocytes both indicate that abnormal astrocytic activity and Ca^2+^ signaling can have negative impact on learning.

How could attenuated astrocytic activity and Ca^2+^ signaling affect learning? Behavioral studies have shown that the astrocytic gliotransmitter D-serine improves spatial memory retrieval [45], spatial reversal memory [53], recognition memory [54, 55], and working memory [54]. Interestingly, we observed that the learning impairments in the motor-skill task caused by blocking astrocytic activity with FC could be rescued by the systemic administration of D-serine on days 6 and 7 of the training. The trend in enhanced performance on days 6 and 7 with D-serine administration in saline injected mice could indicate that increased D-serine levels during motor-skill task facilitate learning (or that D-serine is found in limiting amount in the brain). Since motor-skill learning is thought to be mediated by an NMDA receptor-dependent LTP-like mechanism [36], it is possible that reduced release of D-serine from metabolically inhibited astrocytes prevents LTP induction with motor-skill training and thereby impairs learning in these mice. This interpretation is supported by experiments that demonstrate that D-serine contributes to synaptic plasticity in several brain regions including the hypothalamus, hippocampus, and prefrontal cortex [12–14]. Here we found that FC treatment, completely blocked LTP induction in acute slices from the primary motor cortex. LTP was found to be partially restored by coapplication of D-serine and this rescue was dependent on NMDAR activity. Disrupting Ca^2+^ signaling in astrocytes has been shown to reduce D-serine release [10, 13, 18]. Since exocytosis in astrocytes is controlled by local Ca^2+^ microdomains [56], the reduction in spontaneous Ca^2+^ transients could result in decreased D-serine release from metabolically compromised astrocytes. Indeed disruption of astrocytic metabolism by FC has been shown to decrease Ca^2+^-dependent glutamate release from astrocytes [57]. Our data suggests that astrocytes in the motor cortex have a role in the induction of NMDA receptor-dependent LTP which likely relies on D-ser.

A major molecular mechanism of LTP is the synaptic insertion of GluA1-containing AMPA receptors [58]. Synaptic insertion of GluA1 has been shown with multiple learning paradigms [59–62]. We have recently shown that motor-skill learning results in a transient increase in the synaptic GluA1 in the motor cortex [38]. Here, we find that blocking astrocytic activity with FC interferes with the motor-skill training-induced synaptic incorporation of GluA1 receptors. To our knowledge this is the first demonstration that astrocytic activity is necessary for AMPA receptor insertion into synapses in general and with learning in particular and provides further support that blocking astrocytic Ca^2+^ signaling is likely to interfere with an LTP-like mechanism during motor-skill training.

Motor-skill learning is associated with formation and specific stabilization of dendritic spines [38, 63–65]. Recent studies demonstrate a role for astrocytic perisynaptic processes in regulating dendritic spine stability. Increased neuronal activity, through whisker stimulation, has been shown to enhance motility of perisynaptic astrocytic processes [66, 67], which results in greater synaptic coverage and increased synaptic stability. Activity-dependent enhancement in the motility of astrocytic processes was found to be dependent on astrocytic Ca^2+^ transients and is absent in IP_3_R2 KO mice [67]. It is therefore possible that the perisynaptic astrocytic processes play a role in the stabilization of motor-skill learning-induced new spines by changes in process motility and coverage of the spines and interference with this process lead to the learning impairments we observed in the mIP_3_R2 and FC treated mice.

## Supplementary Material

The supplementary material provides additional information regarding the control groups, experimental design and MRS data.
