# Supplementary material for: Motor-Skill Learning Is Dependent on Astrocytic Activity
Source: Neural Plast. 2015 Aug 4;2015:938023. doi: 10.1155/2015/938023 (PMC4539503; doi:10.1155/2015/938023)
Supplement: Supplementary file 1 — The supplementary material provides additional information regarding the control groups, experimental design and MRS data. [file 938023.f1.pdf]

Supplementary Figures:

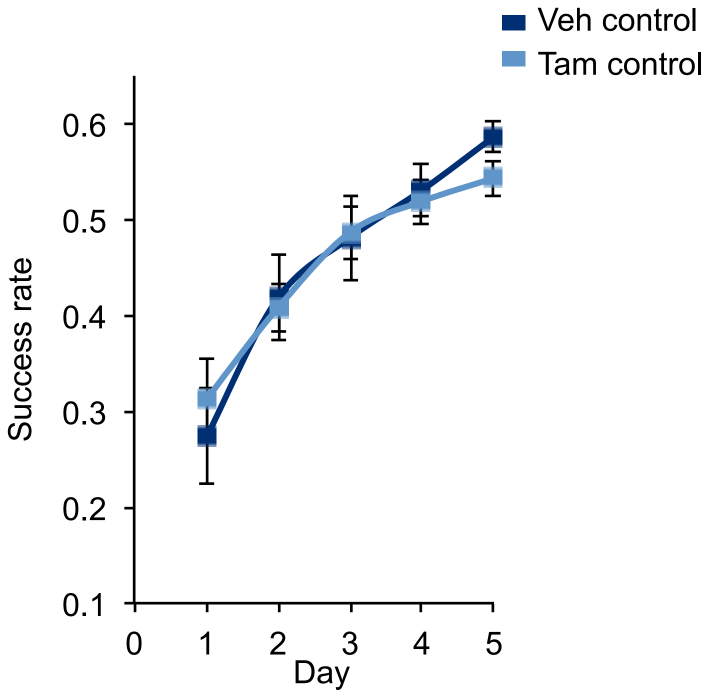

Supplementary Figure 1: **IP_3R2 mediated Ca^{2+} signaling is necessary for motor skill learning.** A. Motor skill learning curves for vehicle control and Tamoxifen control mice. Repeated measures two way ANOVA revealed a significant effect of training ($P<0.0001$) but not genotype ($P=0.848$) and no significant interaction ($P=0.8083$). Vehicle controls versus tamoxifen controls, post-hoc test, P>0.9 for all days of training.

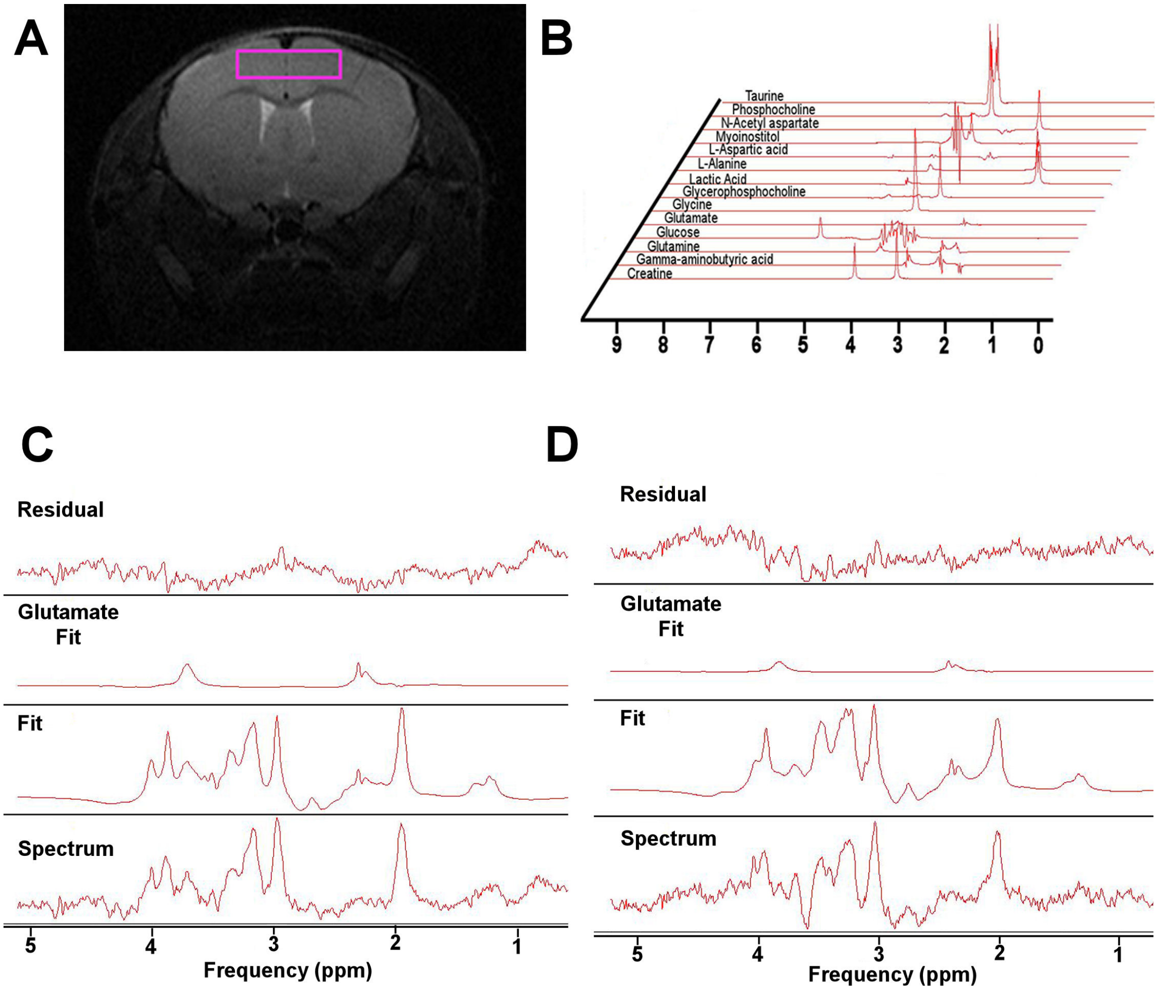

Supplementary Figure 2: Spectroscopic quantitation. A. MRI image demonstrating the region of interest from which spectra were collected. B. Basis set of spectra used for quantitation. Fitting was performed in the time domain skipping the first 12 points to eliminate effects of the baseline (which decays rapidly) from the spectroscopic quantitation. C & D Spectra four hours after the same mouse was injected with either saline (C) or FC (D). From bottom to top is the original spectrum, the fit containing the sum of individual spectra from the basis set, the contribution from glutamate, and the residual baseline after quantitation. Glutamate fit shows that glutamate is decreased after treatment.

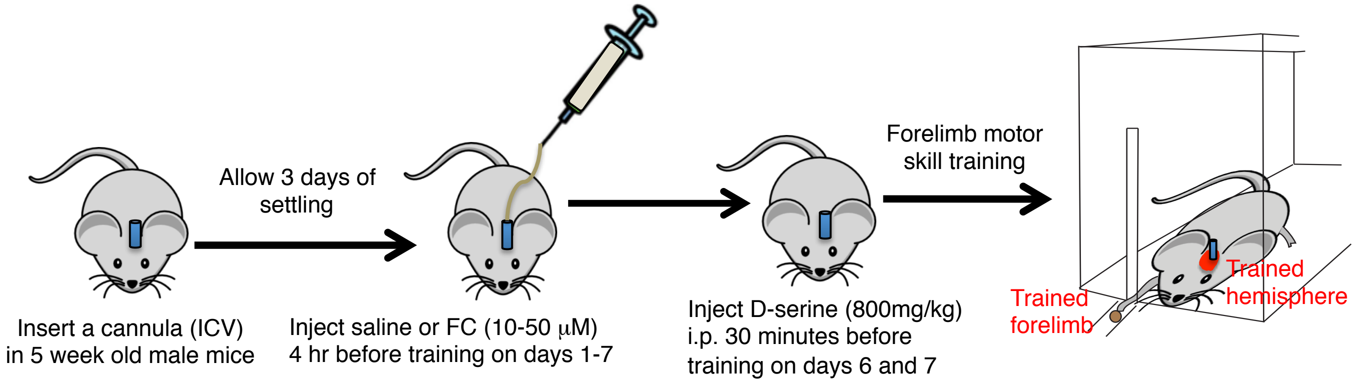

Supplementary Figure 3: Illustration of the motor-skill learning paradigm and the experimental design for FC and D-serine experiments.

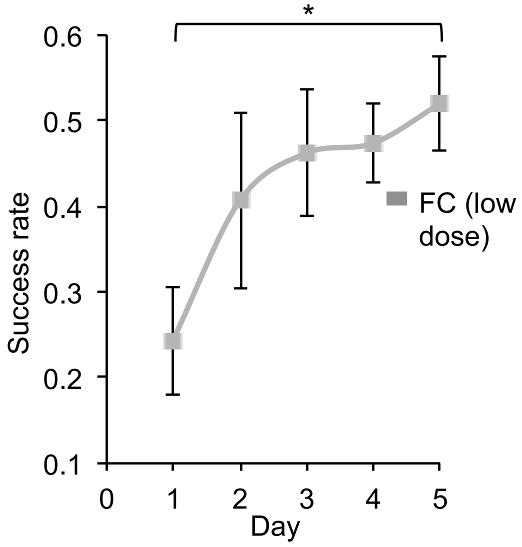

Supplementary Figure 4: Low dose of FC does not interfere with motor skill learning. FC injection (10-50nM) did not cause learning impairments (Day 1: success rate of 0.24 ± 0.06, day 5: 0.52 ± 0.06, *n*= 5 mice, t-test, P=0.011). *P<0.05
